# Vertical profile of ambient VOCs in background region of Southwest China from Mt. Fanjing observation

**DOI:** 10.1038/s41598-026-55939-2

**Published:** 2026-06-10

**Authors:** Dan Yao, Yuan Yang, Hong Hu, Jingting Yang, Haibo Li, Xiangwen Hou, Hao Zhang, Xinmin Zhang, Huanjia Liu, Jiaqi Liu, Yanan Teng, Yujuan Fan, Ke Cheng, Jingyun Liu, Yingchao Yan

**Affiliations:** 1 School of Environment, Key Laboratory of Yellow River and Huai River Water Environment and Pollution Control, Ministry of Education, Henan Key Laboratory of Environmental Pollution Control, Henan Normal University, Xinxiang, Henan China; 2 Guizhou Research and Designing Institute of Environmental Sciences, Guizhou Academy of Environmental Science and Design, Guiyang, China; 3 Fanjingshan National Nature Reserve Administration, Tongren, Guizhou China; 4 Guizhou Fanjingshan Forest Ecosystem Observation and Research Station, Tongren, Guizhou China; 5 School of Public Health, Shandong Second Medical University, Weifang, Shandong China; 6 State Key Laboratory of Environmental Criteria and Risk Assessment, Chinese Research Academy of Environmental Sciences, Beijing, China; 7 College of Energy and Environmental Engineering, Hebei Key Laboratory of Air Pollution Cause and Impact, Hebei University of Engineering, Handan, 056038 China; 8 Inner Mongolia Industrial Technology Engineering Center of Special Resources Development, Utilization and Ecological Environment Protection, Inner Mongolia Normal University, Inner Mongolia, Hohhot, 010022 China

**Keywords:** Volatile organic compounds, Vertical distribution, Source apportionment, Mt. Fanjing, Climate sciences, Environmental sciences

## Abstract

Atmospheric circulation and local meteorology strongly influence the transport and evolution of volatile organic compounds (VOCs) in mountainous environments, yet their vertical distribution and controlling factors in southwest China remain poorly characterized. Here, we conducted synchronous online measurements of VOCs and related air pollutants at the foot (550 m a.s.l.) and top (2119 m a.s.l.) of Mt. Fanjing during autumn and winter 2024, providing a rare side-by-side characterization of VOCs under contrasting atmospheric conditions in the background region of the Yunnan–Guizhou Plateau. The mean VOC concentration at the mountain top was 14.1 ± 4.8 ppbv, 17.5% lower than that at the mountain foot. Source apportionment showed that vehicles, biomass burning, and industrial-related sources together contributed more than 50% of VOCs at the foot site, whereas the relative contributions of aged traffic plume and solvent evaporation increased at the mountain top, reflecting the combined influence of transport and atmospheric processing. Elevated VOC species at both sites were mainly associated with emissions from the border region of Guizhou, Hunan, and Chongqing, in agreement with the emission inventory analysis. Mt. Fanjing was primarily affected by easterly and westerly background winds. Under westerly background conditions, air masses passing through the Sichuan Basin, Chongqing, and Yunnan enhanced the vertical gradient of VOCs at Mt. Fanjing and increased near-surface VOC accumulation, particularly during periods with pronounced diurnal evolution of local circulation. These results demonstrate that the vertical contrast in VOCs over Mt. Fanjing is jointly controlled by regional transport, background wind regimes, and thermally driven local circulation. The findings highlight that elevated mountain sites cannot be regarded simply as clean background environments and have important implications for regional air-quality assessment, ozone and secondary aerosol formation, and pollutant exposure in mountainous and ecologically sensitive areas.

**Supplementary Information:**

The online version contains supplementary material available at 10.1038/s41598-026-55939-2.

## Introduction

As a crucial precursor of ozone (O_3_) and secondary organic aerosols (SOA), volatile organic compounds (VOCs) has attracted sustained attention from scientific and regulatory communities over the past decades due to its profound effects on ecological environment and human health^1–8^. Over the past decades, restrictions on VOC emission sources have been imposed to mitigate high VOC emissions to the greatest extent in China, and since the implementation of the Clean Air Action, the spatial pattern of industrial VOC emissions has shifted, with significant reductions in key regions but notable increases in surrounding areas, for instance, central Guizhou witnessed a 31.8% rise in industrial VOC emissions from 2013 to 2019, contrasting with the overall declining trend in most key regions^9^. This divergence underscores the evolving and heterogeneous spatiotemporal dynamics of VOC emissions between key and non-key areas.

The vertical and horizontal distributions of pollutants play important roles in determining their environmental effects. In situ and remote sensing techniques have been widely used to characterize aerosol vertical profiles^10–14^. Overall, mountain sites are often regarded as proxies for regional background pollution levels, helping to address data gaps in air quality research across China^15,16^. Compared with plain areas, pollutant levels and VOC sources in mountainous regions are more sensitive to elevation and complex terrain related meteorological processes, and these processes can lead to distinct pollutant characteristics between mountain foot and mountain top sites, with mountain foot sites being more affected by local anthropogenic emissions and mountain top sites being more strongly influenced by horizontal transport and vertical exchange^14,17–23^.

Previous studies have showed the great effects of complex terrain and local circulation on pollutant distributions at mountain sites. For example, Shu et al.,^24^ reported higher O₃ concentrations at a mountain top than at the foot, which was attributed to distinct source contributions and transport-dominated mechanisms. Xue,^25^ demonstrated that the combined effects of the Asian monsoon and local valley winds drove the accumulation of O₃ and VOCs at a mountain top, with nocturnal transport across the Qinling Mountains causing rapid O₃ increases of 80–152 ppbv. Similarly, persistent valley wind circulation was shown to elevate VOC concentrations by a factor of 1.5 in the North China Plain^26^, while aerosol levels on Mt. Hua were influenced by daytime valley winds transporting pollutants from the mountain foot to the top^27^.

However, the vertical profile of ambient VOCs in Yunnan-Guizhou Plateau region has been rarely studied, to address these research gaps, this study conducted synchronous measurements of VOCs and O₃ at both the foot and mountain top of Mt. Fanjing during autumn and winter. The objectives are to: (1) elucidate the vertical distribution characteristics and compositional profiles of VOCs; (2) identify and quantify dominant emission sources using receptor modeling and meteorological analysis; (3) revealing the impact of local circulation and regional transport on the vertical exchange of VOCs and (4) provide data support and a scientific basis for formulating air pollution control strategies in southwestern China.

## Results and discussion

### Concentration and compositions of VOCs

**Fig. 1:**
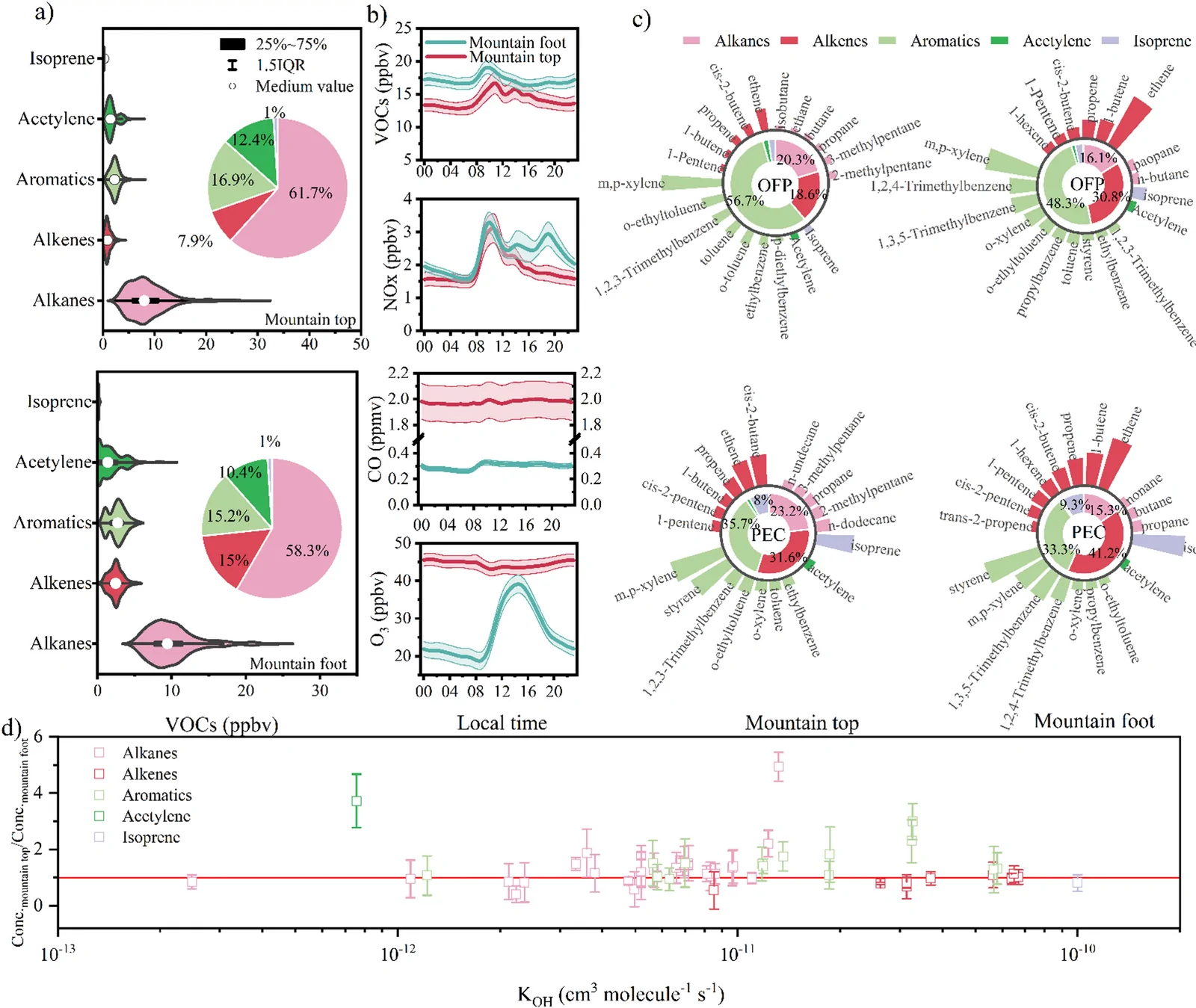
(**a**) Concentration and proportional composition of VOC groups at the two sites. (**b**) Diurnal patterns of VOCs, NOx, CO and O_3_. Lines represent the average values, and shaded areas represent the SDs. (**c**) Proportions of VOC groups (inner ring) and VOC species (outer bar) according to the OFP and PEC at the two sites. The top 20 VOC species are marked on the outer bar. (**d**) Daytime relationship between the mountain top to mountain foot ratio of VOC species and their rate coefficients for reaction with OH.

During the observation period, the mean VOC mixing ratio was 17.1 ± 6.1 ppbv at the mountain foot and 14.1 ± 4.8 ppbv at the mountain top (Fig. S1), indicating a clear decrease in VOC abundance with elevation at Mt. Fanjing. The VOC level at the mountain top was higher than those reported for the North China Plain, the Yangtze River Delta, and central China, but lower than that in southern China, and was comparable to the levels observed at background sites in northwestern China during autumn and winter^25,26,28–30^. At both sites, alkanes were the dominant group, contributing 61.7% (8.8 ± 4.3 ppbv) at the mountain top and 58.3% (10.1 ± 3.5 ppbv) at the mountain foot, followed by aromatics, which accounted for 16.9% (2.4 ± 1.2 ppbv) and 15.2% (2.6 ± 1.2 ppbv), respectively (Fig. 1a). Although the bulk chemical composition was broadly similar at the two elevations, the dominant individual species differed. Ethane, propane, acetylene, ethene, *m/p*-xylene, and 1,2,3-trimethylbenzene were the major species at the mountain top, whereas ethane, propane, acetylene, ethene, and butane dominated at the mountain foot (Table S1). This difference suggests that the mountain-top VOC composition was not simply a diluted expression of near-surface emissions, but also reflected additional atmospheric processing and regional influence.

The diurnal variations further highlight the contrasting characteristics of the two sites (Fig. 1b and Fig. S2). VOC concentrations were generally higher during the day and lower at night at both sites. However, at the mountain foot, VOC levels increased substantially after sunset, leading to a larger foot–top difference during nighttime than during daytime. Total VOCs peaked at 09:00–10:00 at the mountain foot, whereas the maxima at the mountain top were delayed by approximately 1 h for alkanes and alkenes and by about 4 h for aromatics. The delayed peaks at the mountain top imply a weaker direct response to local near-surface emissions and a stronger influence of transport and mixing processes. After sunrise, the nighttime difference in alkane concentrations rapidly decreased, consistent with the development of the boundary layer and enhanced vertical exchange. In contrast, the difference in aromatic concentrations declined sharply after noon of the two sites, reflecting their stronger photochemical reactivity. Alkenes showed persistent differences between the two sites throughout the day, whereas acetylene exhibited only limited inter-site differences except during rush hours.

Source diagnostic ratios supported different controlling factors at the two elevations. The mean toluene/benzene (T/B) ratio was 0.8 ± 0.2 at the mountain top and 0.6 ± 0.3 at the mountain foot, and the T/B ratio at the mountain top showed relatively small diurnal variability (Fig. S3). This feature indicated that the mountain top site was more strongly influenced by regionally transported and relatively well-mixed air masses. By contrast, the larger fluctuations in both T/B and *m*,* p*-xylene/ethylbenzene (X/E) at the mountain foot suggested stronger impacts from local emissions and boundary layer evolution. Moreover, as shown in Fig. S4, the correlations between daytime VOCs (excluding aromatics) and NOx were stronger than those at night, supporting the importance of vertical transport from the mountain foot to the mountain top. However, except for acetylene (*R* = 0.7, *p* < 0.05), the correlations of alkanes and alkenes between the two sites were weak (*R* < 0.5, *p* < 0.05), and aromatics showed no significant inter-site correlation. Overall, the mountain foot was mainly affected by local sources, whereas the mountain top more strongly reflected the integrated influence of vertical transport and regional background air. In addition, the inter-site contrast for individual VOCs also depended on compound-specific atmospheric lifetime and chemical evolution during transport, rather than being determined by source differences alone.

This interpretation was also supported by the behavior of NOx, CO, and O_3_. The mean NOx mixing ratio at the mountain foot (2.4 ± 1.5 ppbv) was about 1.3 times that at the mountain top, and the NOx peak at the mountain top occurred about 1 h later than at the mountain foot (Fig. 1b), consistent with delayed transport from lower elevations. In contrast, CO remained consistently higher at the mountain top and exhibited only weak diurnal variability, suggested that mountain-top CO was not controlled solely by fresh upslope transport from the mountain foot, but also included a more stable background contribution, such as aged air masses, residual-layer air or regional transport. O_3_ showed opposite diurnal cycles at the two sites. At the mountain foot, O_3_ increased during daytime, indicating active local photochemical production. However, O_3_ exhibited a nocturnal maximum and daytime minimum, without an obvious daytime buildup at the mountain top site. Importantly, episodically elevated NO at the mountain top under high O_3_ conditions suggested the presence of local NO emissions near the mountain top. This local source may also affect some VOC species, particularly short-lived compounds, and therefore the mountain top observations likely reflected the combined influence of regional background, vertical transport from lower elevations, and local summit emissions. Although the boundary layer height (BLH) was below 200 m at 08:00 and developed further during the day (Fig. S5), this process mainly affected reactive species on short timescales and does not fully explain the persistently elevated CO and the distinct VOC composition at the mountain top.

The reactivity analysis showed that VOCs at the mountain foot had a stronger capacity to drive local photochemistry than those at the mountain top. The ozone formation potential (OFP) was 119.2 ± 39.3 µg m^− 3^ at the mountain foot and 89.0 ± 48.4 µg m^− 3^ at the mountain top, while the mean propylene-equivalent concentration (PEC) of total VOCs were 5.0 ± 1.7 ppbv and 3.2 ± 1.7 ppbv, respectively (Fig. 1c). Aromatics were the dominant contributors to OFP at both sites, accounting for 48.3% at the mountain foot and 56.7% at the mountain top. The main reactive species included xylene, ethylbenzene, trimethylbenzene, toluene, ethene, propene, and butene. The higher PEC at the mountain foot was mainly driven by elevated xylene and styrene, indicating stronger near-surface photochemical activity. Compared with winter observations in major Chinese cities, the PEC at the mountain foot was lower than that in northern China, comparable to central China, and higher than that in southern China^26,31,32^.

The relationship between the top to foot concentration ratios and OH reaction rate coefficients (k_OH_) further constrained the processes governing the vertical contrast in VOCs (Fig. 1d). The inter-site variability of different compounds was not controlled solely by differences in source influence, but also by their atmospheric lifetime and chemical evolution during transport. In general, species with higher OH reactivity are expected to undergo stronger depletion during upslope transport if photochemical oxidation is an important sink. Consistent with this expectation, several light alkanes and alkenes showed top to foot ratios below unity, indicating substantial chemical loss during transport from the mountain foot to the mountain top. By contrast, relatively long-lived species, such as ethane, propane, acetylene, and benzene, can better survive vertical and regional transport and therefore tended to exhibit smaller inter-site gradients or more coherent temporal variations between the two elevations. This compound dependent behavior suggested that the weaker variability observed at the mountain top partly reflected atmospheric aging during transport. However, some reactive aromatics exhibited ratios close to or even above unity, implying that their abundances at the mountain top were not determined by photochemical removal alone, but were additionally influenced by regional transport, atmospheric processing, and possibly local inputs near the mountain top. This behavior differed from that reported at Mount Tai, where aromatic ratios were generally below unity^33^, suggesting differences in source structure and atmospheric conditions between the two mountain sites. In addition, acetylene and long-chain alkanes (C > 10) showed markedly elevated top to foot ratios (approximately 2.9), indicating that the accumulation of anthropogenic air masses transported on a regional scale also contributed substantially to mountain top VOCs. Isoprene was only slightly lower at the mountain top (ratio = 0.8), suggesting that under low-temperature conditions, local biogenic emissions from mountain vegetation were limited and that regional transport outweighed local biogenic input.

### Influence of meteorological factors on VOCs concentration

**Fig. 2:**
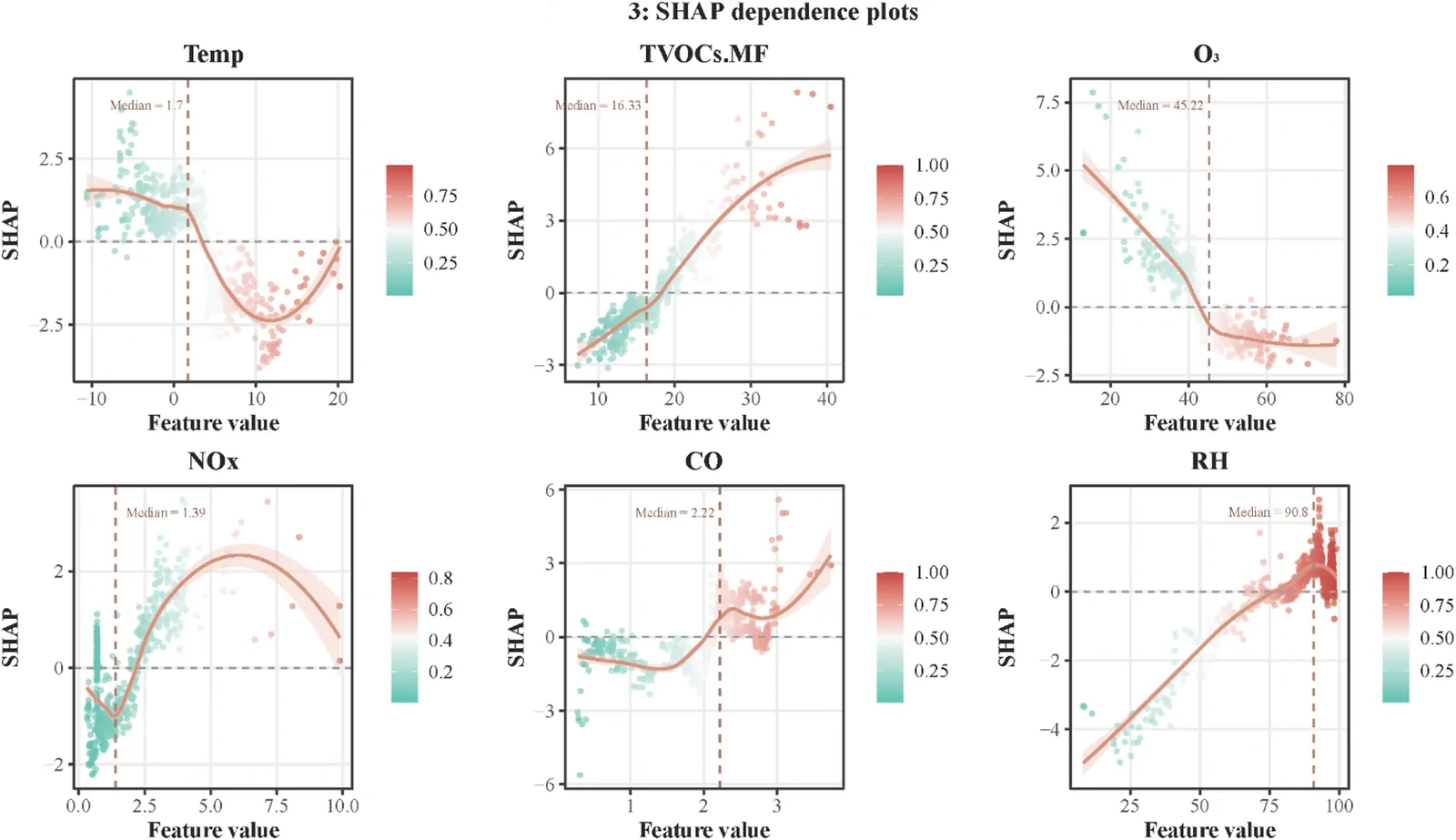
SHAP dependence plots showing the effects of T (Temp), TVOCs at the mountain foot site (TVOCs.MF), O_3_, NOx, CO, and RH on TVOCs at the mountain top site.

The eXtreme Gradient Boosting (XGBoost) model from both global contribution and local response perspectives revealed the main driving factors of VOC variation at the mountaintop site and their effect directions (Fig. 2, Fig.S6). The results showed that temperature (Temp), TVOCs.MF, and O_3_ were the key variables affecting mountaintop VOCs, with contribution rates of 17.8%, 17.2%, and 16.1%, respectively. These were followed by NOx, CO, and relative humidity (RH), contributing 13.1%, 13.0%, and 11.5%, respectively. The cumulative contribution of the top six variables reached 88.7%, indicating that mountaintop VOCs were mainly influenced by the combined effects of meteorological conditions and coexisting pollutants, whereas pressure, wind direction, and wind speed played relatively minor roles.

In terms of effect direction, TVOCs.MF, CO, and RH generally showed positive effects on mountaintop VOCs, meaning that higher values of these variables were usually associated with higher predicted concentrations. In contrast, temperature and O_3_ exhibited overall negative effects, suggesting that higher temperature and elevated O_3_ levels were unfavorable for the accumulation of mountaintop VOCs. The SHAP values of NOx were distributed on both the positive and negative sides, indicating a pronounced nonlinear influence on mountaintop VOCs and possible complex interactions with other environmental factors. Further SHAP dependence analysis showed that the suppressive effect of temperature on mountaintop VOCs was more pronounced in the medium-to-high temperature range. The positive effects of TVOCs.MF and RH gradually strengthened with increasing values, whereas O₃ consistently showed a negative effect. Both NOx and CO displayed threshold characteristics: they mainly contributed negatively at low levels, but gradually shifted to positive contributions and became markedly stronger once a certain threshold was exceeded. Overall, the variation in mountaintop VOCs was not controlled linearly by any single factor, but rather resulted from the combined effects of meteorological background and multiple coexisting pollutants, with significant nonlinear response features.

Elevated VOCs concentrations at the mountain top were associated with high (RH > 60%) and moderate temp. (-10 to 8 °C), while similar conditions (RH > 60%, T = 0 ~ 16 °C) favored accumulation at the foot site (Fig. S7). High RH likely suppressed the oxidative degradation and diffusion of VOCs at the top site, thereby increasing concentrations; conversely, lower RH often coincided with better dispersion. Although higher temperatures generally promote atmospheric diffusion, this study observed high VOCs concentrations at temperatures below 8 °C. Han et al.^34^ reported that high VOCs mixing ratios was observed during heavy pollution episodes with relatively low temperatures (-4 to 5 °C) and high RH (60%), indicating that moderate humidity and temperature conditions are more conducive to VOCs accumulation under typical diffusion scenarios.

### The emission sources of Ambient VOCs

**Fig. 3:**
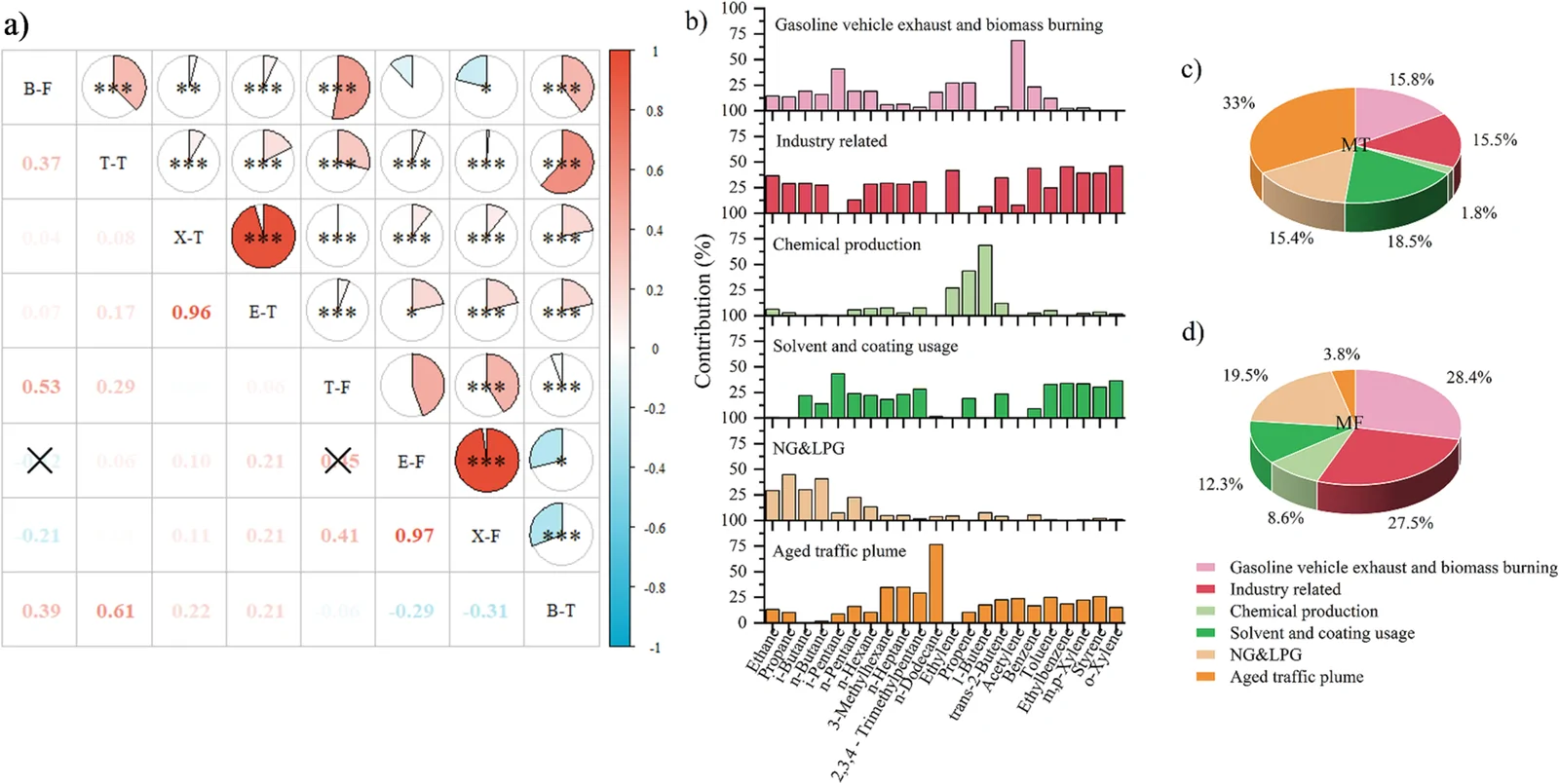
The correlations of benzene (B), toluene (T), ethylbenzene (E), and m/p-xylene (X) are illustrated for the mountain top (T) and foot (F) samples, the size and color of the circles represent the relative strength of the correlations, and the numbers indicate the specific values, ***p < 0.01, *p < 0.05 (**a**); Source identification based on the result of PMF (**b**), and (**d**) the contribution of each emission sources at the mountain foot (MF) and (**c**) mountain top (MT) site during the observation period.

Vertical diffusion of pollutants was evidenced by elevational delays in contaminant peaks and decreasing reactive species concentrations with height, implying common emission sources contribute to VOCs at both top and foot sites. During the observation period, strong positive correlations between ethylbenzene (E) and m/p-xylene (X) were observed at each individual site (Fig. 3a). In contrast, correlations for the same VOC species between the top and foot sites were weak, indicating significant differences in VOC chemical composition between the two elevations due to varying source influences. Due to the relatively low emissions of biogenic sources in autumn and winter, and isoprene (the biogenic tracer observed in this study only included isoprene, and observations of secondary products were lacking) is easily oxidized to form secondary products during transport. Therefore, the sources of VOCs in this study mainly focused on anthropogenic emissions.

Positive Matrix Factorization (PMF) results identified six major emission sources including gasoline vehicle exhaust & biomass burning, industrial-related emissions, chemical production, solvent and coating usage, natural gas (NG) & liquefied petroleum gas (LPG) emissions, and aged traffic plume during the observation period (Fig. 3ba). Apparently, the source contributions differed markedly between the two sites (Fig. 3c, d), among them, gasoline vehicle exhaust, biomass burning, and industrial-related sources contributed over 50% of the total VOCs at the mountain foot site. In contrast, the mountain top was predominantly influenced by aged traffic plume, and solvent usage constituted a substantially larger fraction of VOC loading at the mountain top than the mountain foot site, indicating the mountain top site were more likely reflected anthropogenic plumes transported from lower-elevation source regions and subsequently modified by atmospheric mixing and chemical aging during transport.

As high loads of toluene, acetylene, and m/p-xylene were observed at the two sites, and they are typical tracers for solvent usage, vehicle exhaust, and coal/biomass combustion, they were selected for Concentration-Weighted Trajectory (CWT) analysis to identify potential source regions influencing the Mt. Fanjing receptor sites, and the method of CWT model has been detailed described by Xue et al.,^25^. In the CWT model, concentrations exceeding the 75th percentile of the dataset (toluene: 0.34 ppbv, acetylene: 1.68 ppbv, m/p-xylene: 0.64 ppbv at the mountain top; toluene: 0.34 ppbv, acetylene: 2.61 ppbv, m/p-xylene: 0.56 ppbv at the mountain foot) were classified as “polluted”. Higher CWT values indicated a greater probability that an air parcel passing through a grid cell resulted in high measured concentrations. As shown in Fig. S8, five distinct air mass pathways were identified as major contributors to VOC loading at the top within 24 h: (a) short-distance transport from the northwest, northeast, and southeast within Guizhou Province; (b) short-to-medium and medium-range northeasterly air masses originating from Hunan Province and passing through Chongqing; and (c) long-range transport of northeasterly air masses from southern Hunan Province. In contrast, four air mass types influenced the foot site, with northeastern transport patterns consistent with the mountain top, though short-distance contributions originated mainly from northeastern Guizhou.

**Fig. 4:**
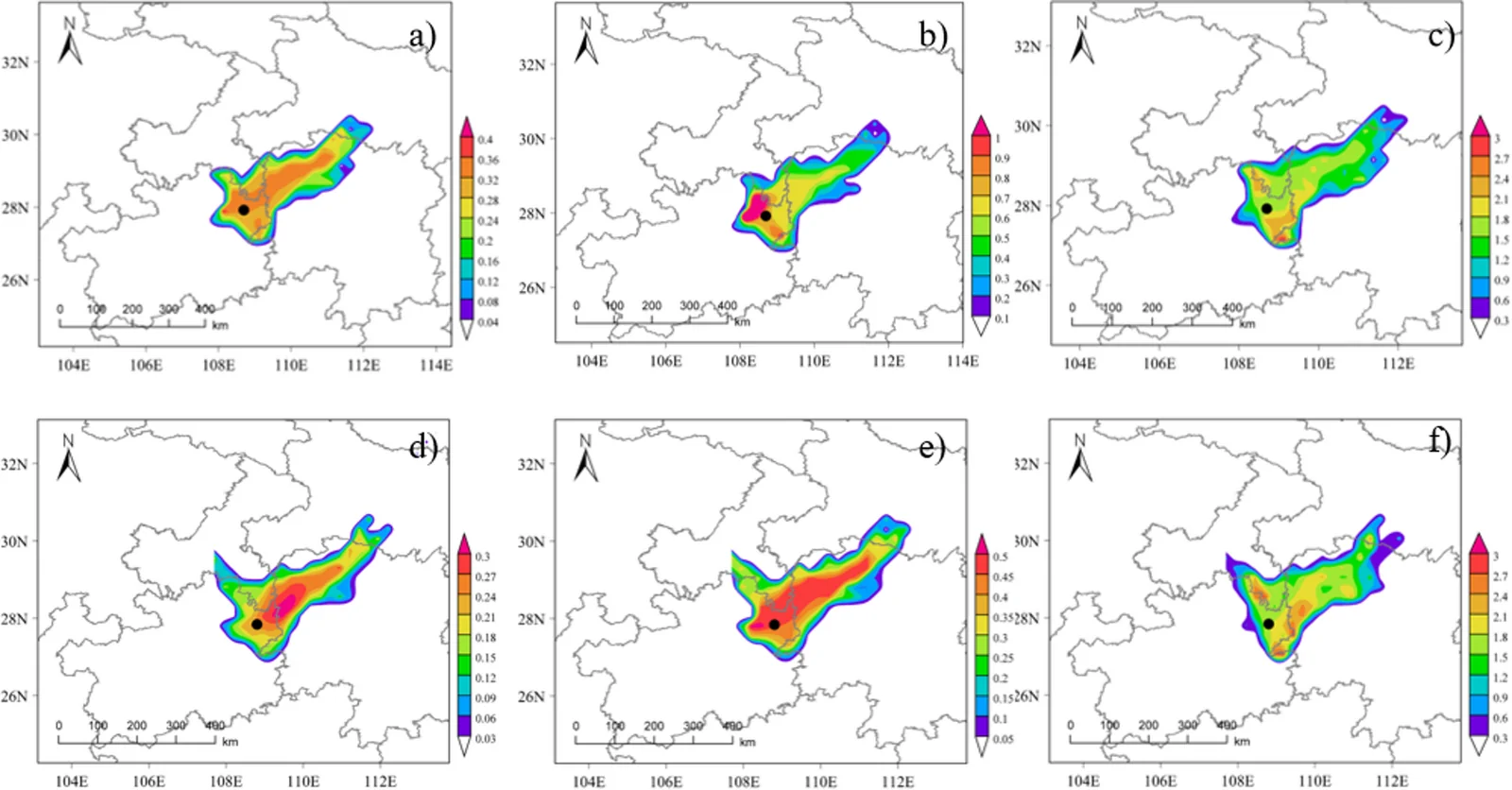
Potential source distribution of toluene (**a**), m/p-xylenes (**b**), acetylene (**c**) at the mountain top site, and toluene (**d**), m/p-xylenes (**e**), acetylene (**f**) at the mountain foot site.

Throughout the observation period, northeastern air flows dominated regional transport (Fig. 4). The spatial distributions of potential sources for toluene, acetylene, and m/p-xylene exhibited significant differences. At the mountain top, high CWT values for toluene were concentrated in northwest Hunan Province, high loads of acetylene and m/p-xylene levels were largely attributable to the transport from Guizhou Province, indicating the local emissions in Tongren and surrounding areas were the key potential sources of acetylene and m/p-xylene, besides, high CWT values were also identified along northeasterly trajectories passing through Chongqing and western Hunan Province. At the foot site, potential source regions for toluene and m/p-xylene were similarly distributed, with high CWT values located near the Hunan–Guizhou–Chongqing border. Acetylene source patterns were more consistent with those at the mountain top site. Compared with the mountain foot site, the mountain top patterns were broader and smoother, which was more consistent with regional transport, atmospheric mixing, and aging during transport.

According to the source list statistics results (Multi-resolution Emission Inventory for China, MEIC), civil coal combustion was the largest source of VOC emissions in Guizhou and Hunan province, and the annual VOC emissions of civil coal combustion continued to increase from 2010 to 2020. Solvent usage including industrial coating, printing & dyeing and architectural coatings accounted 18.8%~32.7%, 24.4%~47.3% and 8.4%~20.3% in Hunan Province, Chongqing and Guizhou Province, respectively. In addition, the annual average emissions of VOCs related to vehicles had decreased, but the overall emissions of gasoline vehicles, petrochemicals, oil and gas storage and transportation still showed high levels. Overall, the emission inventory suggested that residential combustion, solvent use, and vehicle-related sources were important anthropogenic VOC source categories in the border areas among Hunan, Chongqing, and Guizhou. Combined with the observed VOC composition and source apportionment results, these regions were considered plausible upwind source areas influencing Mt. Fanjing during autumn and winter.

### Vertical profile of VOCs with the air mass transport

Vertical exchange and horizontal transport influence the vertical distribution of ambient VOCs concentrations^23,35–38^. In the Mt. Fanjing region, low-level background winds are characterized by both east-to-west and west-to-east transport. The east-to-west flow indicates a reversal of the prevailing transport pattern, while the west-to-east flow is primarily driven by the zonal westerly belt and transport along the edge of the Plateau.Taking into account the local circulation and the day–night differences in valley winds/slope winds over Mt. Fanjing on the observation days (Fig. S6), the observation period were mainly divided into easterly background circulation regime–weak local diurnal variation circulation (E-weak), easterly background circulation regime–moderate local diurnal variation circulation (E-moderate), easterly background circulation regime–pronounced local diurnal variation circulation (E-strong), westerly background circulation regime–weak local diurnal variation circulation (W-weak), westerly background circulation regime–moderate local diurnal variation circulation (W-moderate), westerly background circulation regime–pronounced local diurnal variation circulation (W-strong), the screening criteria and specific dates are provided in Text S1 and Table S2 of the Supplement information.

**Fig. 5:**
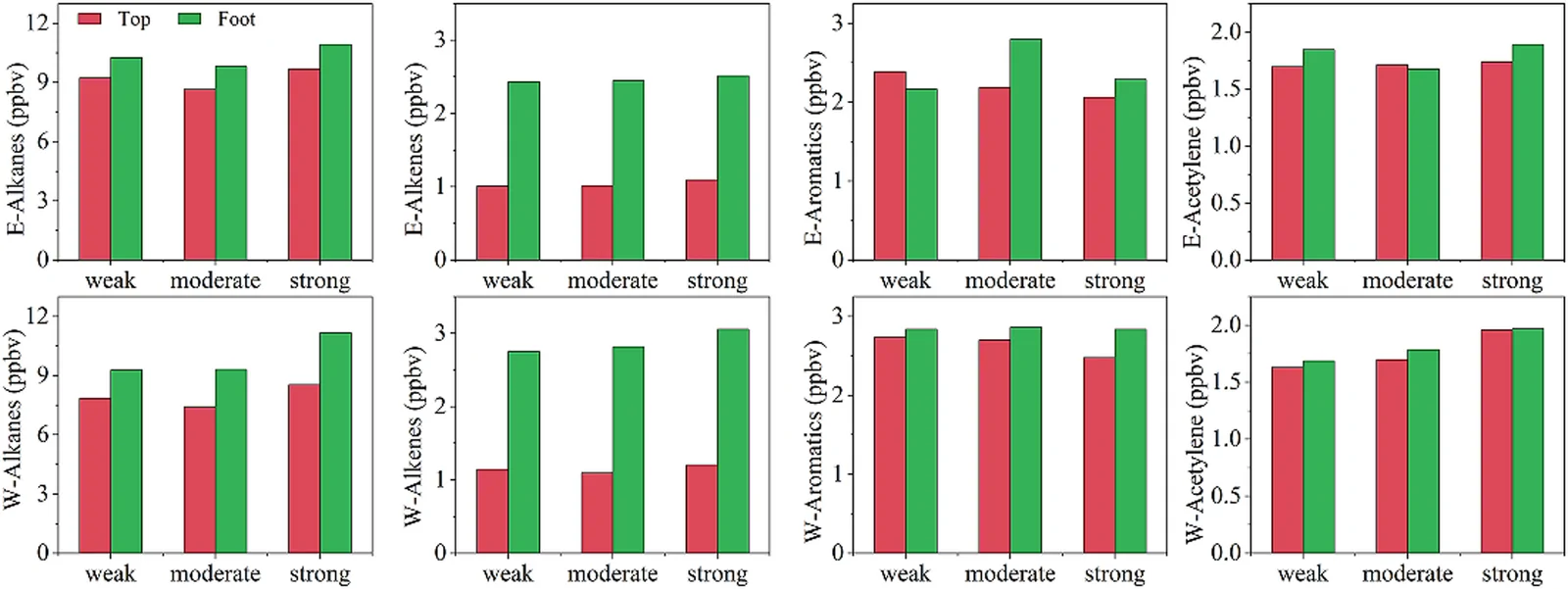
Differences in the concentrations of various VOCs between the mountaintop (Top) and the foot of the mountain (Foot) under different background and local circulation conditions.

As shown in Fig. 5, under all combinations of background and local circulation, VOC concentrations at the mountain foot were consistently higher than those at the top site. When the flow was dominated by an easterly background circulation, the discrepancy of VOC concentrations between the top and the foot ranged from 2.4 to 3.1 ppbv, whereas under a westerly background circulation the difference increased to 3.1 ~ 4.8 ppbv, indicating stronger VOC accumulation at the foot of Mt. Fanjing under westerly conditions, especially during the periods with pronounced local diurnal variation. Moreover, under the easterly background regime, the impact of enhanced local diurnal variation on total VOC concentration was relatively weak, with total VOC concentration at the top remaining within a narrow range of 13.6 ~ 14.5 ppbv, and only a slight increase under the “pronounced diurnal variation” regime at the foot, suggesting that valley/slope winds mainly regulate vertical redistribution rather than substantially modifying the overall regional VOC burden. In contrast, under the westerly background regime, total VOC concentration exhibited a much more pronounced increasing trend with intensifying local diurnal variation circulation, with total VOC concentration increasing from about 13.4 to 14.2 ppbv at the mountain top and total VOC concentration at the foot rising markedly from approximately 16.5 to 19.0 ppbv, accompanied by a substantial increase in alkanes and moderate increases in alkenes and acetylene. Particularly under the “W-strong” regime, both total VOC concentration and the levels of highly reactive species (such as alkenes and some aromatics) at the foot reached the highest values, implying that the superposition of large-scale westerly transport and strong valley/slope circulations favors VOC accumulation rather than dilution within the valley. The results highlight the crucial role of background wind patterns and local thermally driven circulations in regulating the vertical distribution and near-surface build-up of VOCs in the Mt. Fanjing region.

**Fig. 6:**
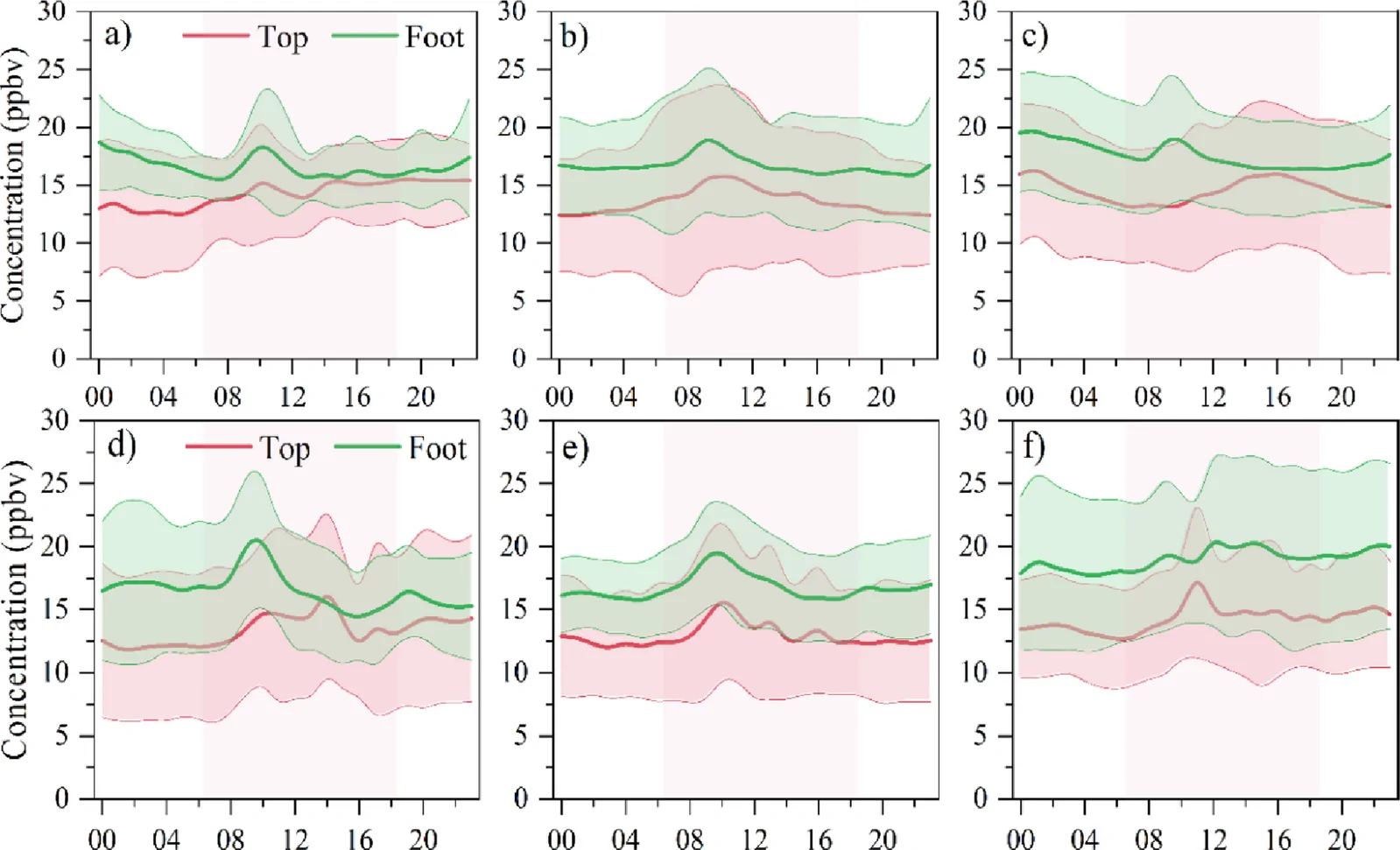
Diurnal VOCs variation at the mountain top (Top) and mountain foot (Foot) sites during (**a**) E-weak, (**b**) E-moderate, (**c**) E-strong, (**d**) W-weak, (**e**) W-moderate, (**f**) W-strong, the average sunrise and sunset times during the observation period are 6:30 and 18:30, respectively. The shaded area represents the daytime observation period.

Overall, VOC concentrations at the mountain foot site were systematically higher than the mountaintop site, but the magnitude of this difference and its diurnal evolution vary markedly with the background circulation and the strength of local diurnal circulations (Fig. 6). When the lower-level background over the Mt. Fanjing region was influenced by westerly winds, the background VOC concentration was relatively low, whereas the foot site exhibited distinct VOC accumulation, especially during periods with pronounced diurnal variation of local circulations. In contrast, when the low-level background was affected by easterlies, nighttime background concentrations increased to varying degrees, while VOC accumulation at the foot was relatively weak. When the diurnal variation of local circulations was weak, regional VOC concentrations were mainly controlled by large-scale background circulation, and the vertical VOC concentrations difference decreased gradually during the afternoon (to 0 ~ 2.1 ppbv and 0.9 ~ 2.7 ppbv), but increased again at night, and the timing of the VOC diurnal peak differs little with height (around 10:00), indicating there was pronounced vertical exchange of VOCs during the daytime as the mixed layer develops with the influence of large-scale circulation. When the diurnal variation of local circulations reached a moderate level (especially under westerly background conditions), stronger valley winds and their coupling with the background wind during the day facilitated the transport of pollutants from surrounding source regions and the residual layer into the observation area and their retention within the valley/slope system, thereby increasing daytime VOC levels by about 0.1 ~ 2.3 ppbv, down slope winds and a weakened stable layer favored the removal and dilution of near-surface pollution, reducing nighttime VOC accumulation by about 0.1 ~ 2.0 ppbv, so that the “high by day and low by night” feature became more pronounced compared with periods of weak local circulations.

In addition, when the diurnal variation of local circulations was strong, VOC concentrations at the mountain foot showed a marked accumulation, with a maximum increase of up to 5.6 ppbv at 15:00, while the regional background VOC concentrations also increased to some extent during 00:00 ~ 06:00 and 12:00 ~ 16:00. A possible explanation was continuous regional-scale combustion emissions (consistent with the relatively small fluctuations in acetylene) at night (Fig. S7), the presence of a low-level jet or weak background winds can slowly transport polluted air masses from upwind source regions to the airspace over the study area; the weak ventilation leads to gradual accumulation in the background layer and thus a slight increase in background VOCs before dawn. In the afternoon, enhanced valley winds continuously transport high concentration air masses from surrounding source regions into the valley and mountain foot areas, where the complex terrain favored the re-circulation and trapping of these air masses, while the valley winds and the rising boundary layer help mix VOCs accumulated in the valley and in downwind urban areas into the larger-scale background layer.

**Fig. 7:**
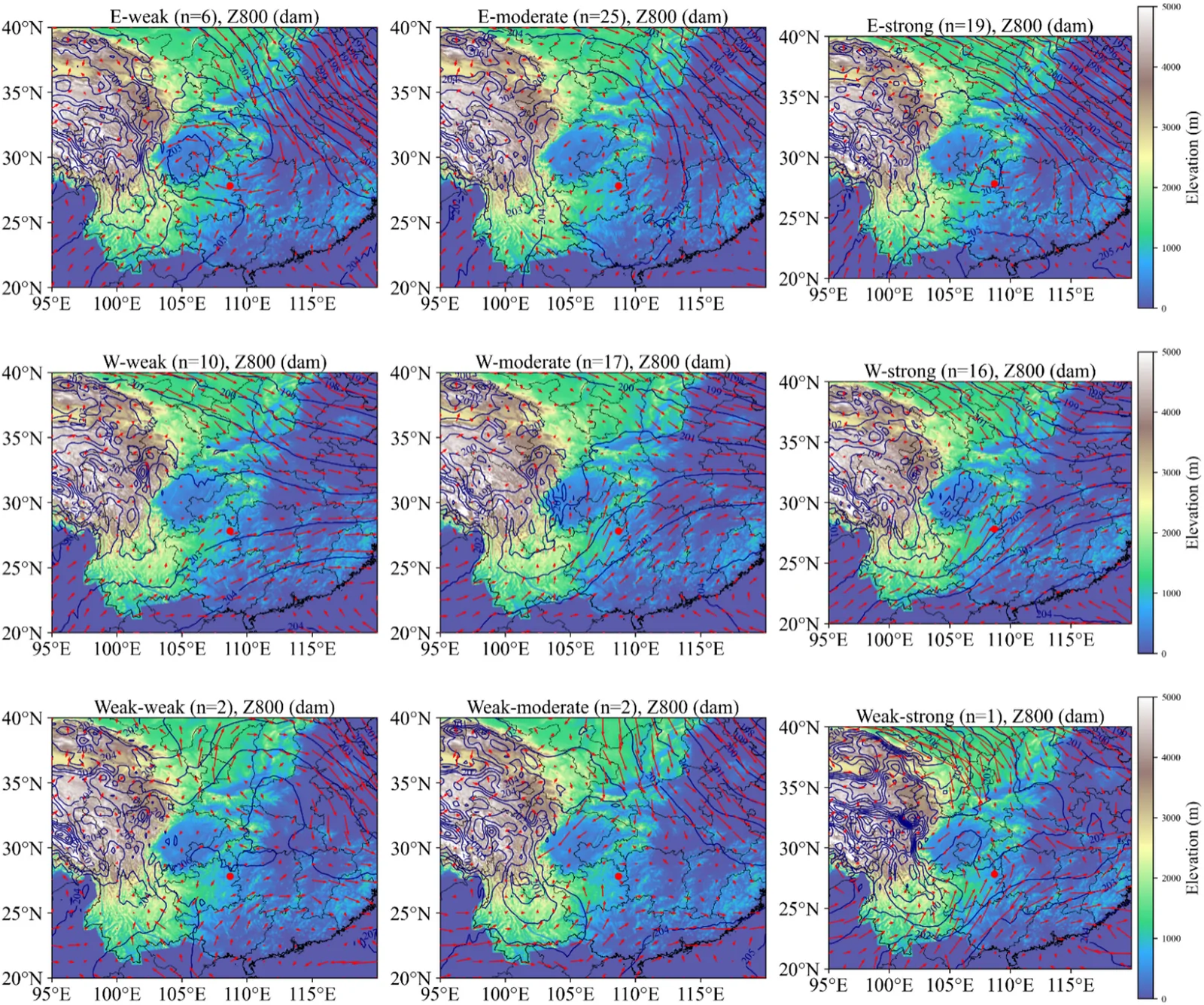
The average of pressure contour and horizontal winds at a height of 800 hPa in the area surrounding Mt. Fanjing during (n represent the days, the data was download by https://cds.climate.copernicus.eu/datasets/reanalysis-era5).

In addition, as shown in Fig. 7, during the observation period, the lower atmosphere over Mt. Fanjing was predominantly influenced by air masses transported from east to west (i.e., reverse transport), with circulation regimes mainly characterized by moderate diurnal variations in local circulations. When the background wind was easterly, the center of the western Pacific subtropical high was located to the east of Mt. Fanjing, and low-level air masses were transported along the western flank of the subtropical high from the northwestern Pacific, across the East China Sea/South China Sea and the coastal regions of eastern and southern China, and then inland over central China before reaching Mt. Fanjing. The westward extension of the subtropical high enhanced this reverse transport, so that the mountaintop was primarily affected by relatively clean background air associated with the subtropical high. As the local circulation intensified, pollutants near the mountain foot were more easily lifted to higher altitudes by valley and slope winds, but were continuously diluted by the prevailing easterlies, resulting in a limited vertical contrast.

In contrast, under westerly background conditions, Mt. Fanjing was embedded in the westerly flow along the eastern flank of the Tibetan Plateau, and low-level air masses were mainly transported eastward along this corridor from upstream source regions over the Tibetan Plateau, the Sichuan Basin, the Chengdu–Chongqing region, and the upper reaches of the Yangtze River to Mt. Fanjing. The dense 800 hPa height contours indicate enhanced large-scale transport and ventilation, leading to persistent flushing of VOCs at the mountaintop and maintaining relatively low concentrations there, whereas topographic blocking and the convergence of valley winds caused pollutants from both upstream and local sources to accumulate near the mountain foot, forming a pronounced vertical gradient with lower VOC concentrations at the mountain top and higher values at the foot under westerly background conditions.

## Conclusions

This study provides a rare side-by-side characterization of VOCs at the mountain foot and mountain top under contrasting atmospheric conditions in the background region of the Yunnan-Guizhou Plateau. The results demonstrated distinct influences of local emissions and long-range transport at the two elevations. The stronger influence of local emissions near the surface resulted in average VOC and NOx concentrations at the foot site being 1.2 and 1.3 times those at the top site, respectively. Alkanes constituted the dominant VOC group at both sites, and the combined influence of small-scale service activities, long-range vehicle emissions, and solvent use led to a slightly higher relative contribution of aromatics at the mountain top than at the mountain foot. The notably high daytime concentration ratio of acetylene and long-chain alkanes (C > 10) between the mountain top and foot (2.9) further underscores the importance of long-range transport to the mountain top. Local circulation and meteorological conditions also emerged as critical drivers of VOC accumulation, with moderate humidity and temperature ranges significantly promoting increases in ambient VOC concentrations.

Regional transport from northwestern Hunan and northeastern Guizhou constituted the primary source of elevated key VOC species at both monitoring sites. Furthermore, when the low-level background winds over the Mt. Fanjing region were generally consistent with the east–west westerly belt, the background concentration at Mt. Fanjing was greatly diluted, whereas background winds opposite to the east–west westerly belt more easily increased the background concentration. In contrast, when the diurnal variation of local circulation was pronounced, the day–night evolution of the wind field became more clearly defined, and daytime valley winds readily transported locally emitted pollutants into the valley region, leading to continuous accumulation of VOCs. Overall, this study provides the first detailed vertical characterization of ambient VOCs in the background region of the Yunnan-Guizhou Plateau and demonstrates that elevated mountain sites in southwest China cannot be simply regarded as clean background environments. Instead, their atmospheric composition reflects the combined influence of local emissions, regional input, and atmospheric processing.

The findings offer critical insights into the vertical distribution, source contributions, and meteorological modulation of VOCs in complex mountainous terrain, thereby providing a scientific basis for improving regional air-quality models and developing more targeted pollution-control strategies in southwest China. They also have implications for understanding pollutant exposure in mountainous and ecologically sensitive areas, as well as the role of VOC oxidation in O_3_ and SOA formation. Future work should extend the present two-site observations to multi-season and vertically resolved measurements, together with more comprehensive characterization of oxidation chemistry, to better constrain the relative roles of emissions, transport, and atmospheric aging in shaping mountain VOC distributions.

## Materials and methods

### Sampling location description

**Fig. 8:**
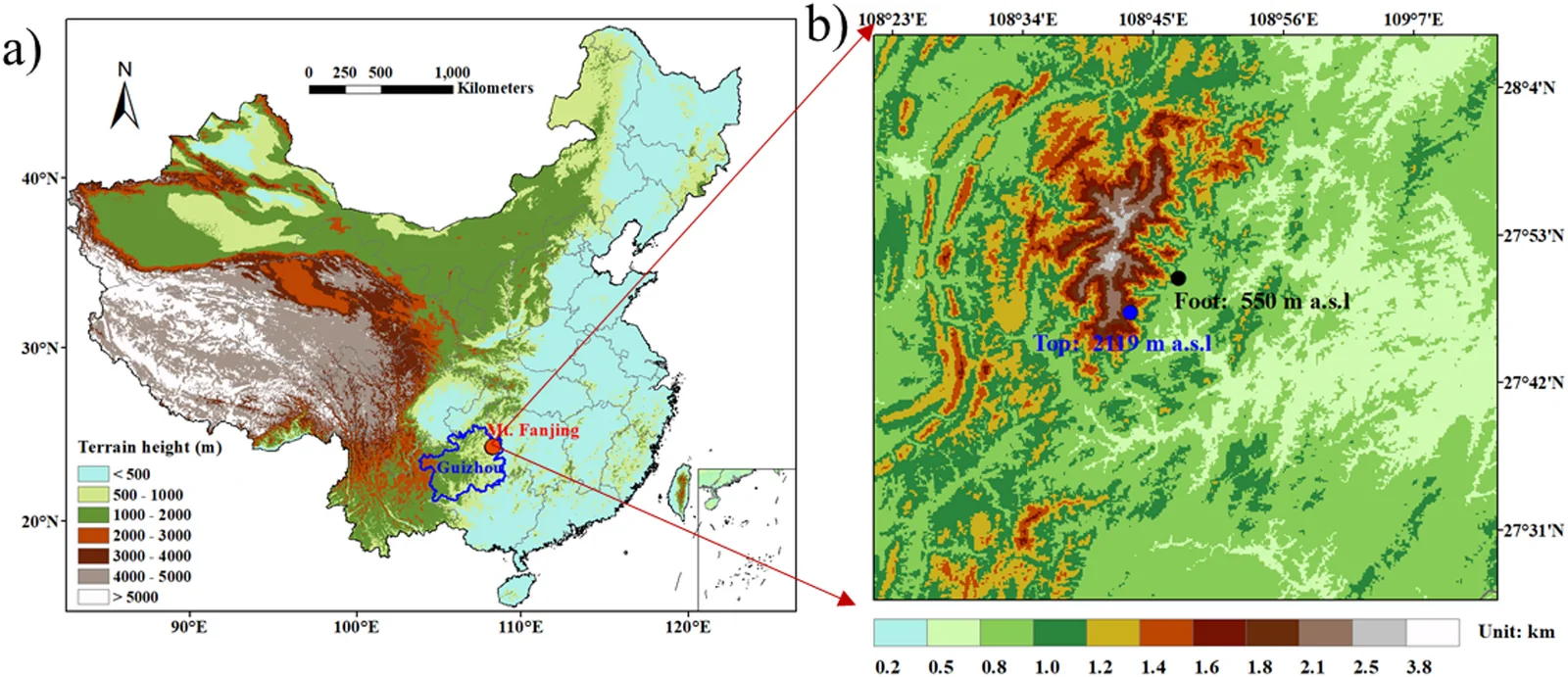
(**a**) Map of Mt. Fanjing in China, and the location of Guizhou marked; (**b**) enlarged area shows the topography of Mt. Fanjing. The locations of the mountain foot (550 m a.s.l) and the mountain top sites (2119 m a.s.l) are marked with solid dots in black and blue, respectively. The maps were generated using ArcGIS software (ArcMap version 10.8.1; Esri, https://www.esri.com/en-us/arcgis/products/arcgis-desktop/overview).

Simultaneous online measurements of VOCs were conducted at a mountain foot site (Heiwanhe; 27°50′45″N, 108°46′3.87″E; 550 m above sea level, a.s.l.) and a mountain top site (upper cable car station; 27°54′36.59″N, 108°41′44.50″E; 2119 m a.s.l.) of Mt. Fanjing from November 2024 to February 2025. The two sites form a typical mountain-foot to mountain-top observational transect with a large elevation difference, which is well suited for examining the vertical variability of VOCs under mountain background conditions. As illustrated in Fig. 8, both sites are located away from major urban and industrial emission centers, and thus provide a useful case for characterizing VOC gradients in the background mountainous regions of Southwest China. Nevertheless, as a nationally renowned scenic area, both sites are subject to specific local emission influences. The foot station is adjacent to the eastern gate of the Mt. Fanjing Scenic Area, with homestays and restaurants located to the south and an eco-friendly sightseeing vehicle route approximately 150 m to the east. The top station is situated at the Wanbaoyan viewing platform near the upper cable car station, with visitor amenities such as cafes and convenience stores within a 200 m radius.

### VOCs and other air pollutants analyze

Ambient VOCs were measured using an automated monitoring system (Ntrace VsCMS6500; Nutech (China) Co., Ltd., Changsha, China). This system provides online quantification of C₂–C₁₂ VOCs, encompassing the 57-PAMS (Photochemical Assessment Monitoring Stations) species. A detailed instrument description is available in the Supplement information (Text S2). Prior to the campaign, a co-location intercomparison was performed, demonstrating strong agreement between identical instruments, with calibration slopes > 0.9 and correlation coefficients > 0.95 for target compounds. Throughout the monitoring period, external standard calibration (0.5, 2, 4, 6, 8, and 10 ppbv) was conducted every second day to ensure quantification accuracy, maintaining relative analytical deviations within 15%.

Co-pollutants were measured with dedicated analyzers compliant with Chinese national standards: O₃ was measured via UV absorption spectrometry (Model EAQM-3000; Skyray Instrument, Jiangsu). NO and NO₂ were detected using a chemiluminescence analyzer (Model EAQM-2000; Skyray Instrument). CO concentrations were determined by non-dispersive infrared spectroscopy (Model EAQM-4000; Skyray Instrument). Meteorological parameters (Temp, RH, pressure, wind direction, and wind speed) were recorded by an automated weather station (Model WZZ-M6; Weizhihuan Energy Technology, Jiangsu). All measurements adhered to China’s National Environmental Protection Standards, with quality assurance and control (QA/QC) procedures meeting the Class I monitoring station specifications stipulated by the Ministry of Ecology and Environment. The emission inventory data used in this study were obtained from the MEIC (http://meicmodel.org.cn/), and the development and methodology of the MEIC inventory have been described in detail by Li et al.^39,40^.

### Propene equivalent concentration and ozone formation potential

The photochemical reactivity of individual VOCs and their potential to form O₃ vary significantly. To assess these aspects, the OFP and PEC were calculated. The OFP for each VOC species i was quantified using the Maximum Incremental Reactivity (MIR) scale^41^, as shown in Eq. (1).

1 $${\mathrm{OFP}}\left( {\mathrm{i}} \right) = {\mathrm{C}}\left( {\mathrm{i}} \right) \times {\mathrm{MIR}}\left( {\mathrm{i}} \right)$$

The PEC, which normalizes the concentration of a VOC to the OH reactivity of propene^42^, was calculated using Eq. (2):

2 $${\mathrm{PEC}}\left( {\mathrm{i}} \right) = {\mathrm{C}}\left( {\mathrm{i}} \right) \times [k_{{{\mathrm{OH}}}} \left( {\mathrm{i}} \right)/k_{{{\mathrm{OH}}}} ({\mathrm{C}}_{{\mathrm{3}}} {\mathrm{H}}_{{\mathrm{6}}} )]$$

Here, C(i) is the measured concentration of species i (ppbv), *k*_OH_ (i) is the reaction rate coefficient of species i with the OH radical, and *k*_OH_ (C₃H₆) is the rate coefficient for the reaction of propene with the OH radical. The MIR coefficients and OH reaction rate coefficients were taken from Carter^43^ and Atkinson et al.^44^, respectively.

### XGBoost and PMF model

In this study, XGBoost model was employed to quantify the influence of meteorological conditions, air pollutants and mountain foot VOC concentrations on mountaintop VOCs. In this study, we employed an ensemble method that leverages bootstrap aggregation and randomized feature selection to robustly capture nonlinear relationships while mitigating overfitting, and used the SHapley Additive exPlanations (SHAP) framework to interpret the model, the theory-based approach has been provided by previous studies^45,46^. The model was trained on 1471 observational datasets, with 678 independent measurements reserved for validation. The detailed description of XGBoost model was provided in Text S4 in the supplement information.

For source apportionment, the US EPA PMF 5.0 model was applied. A total of 22 VOC species (11 alkanes, 4 alkenes, acetylene, and 6 aromatics) were input into the model. Over 90% of the concentration data for these species were above the method detection limit. The uncertainty estimation, detailed model setup, and species selection principle followed established methodologies, as provided in the Supplement information (text S3 and table S3). A six-factor solution was identified as optimal (Q_robust_/Q_true_=0.98), with 96% of the species residuals falling within the range of -3 to + 3 and a bootstrap mapping concordance exceeding 80% (tables S4-S5).

## Supplementary Information

Below is the link to the electronic supplementary material.

Supplementary Material 1

## Data Availability

The dataset generated and analyzed during this study has now been deposited in a public repository. It is openly available (https://doi.org/10.5281/zenodo.18637640, Dan et al., 2026).Dan, Y., Yuan, Y., Hong, H., Jing, T.Y., Hai, B.L., Xiang, W.H., Hao, Z., Xin, M.Z., Huan, J.L., Jia, Q.L., Yan, A.T., Yu, J. F., Ke, C., Jing, Y.L., Ying, C.Y.: Vertical profile of ambient VOCs in background region of Southwest China: insights from Mt. Fanjing observation campaign [datasets], 2026, https://doi.org/10.5281/zenodo.18637640.
