# Supplementary material for: Vertical profile of ambient VOCs in background region of Southwest China from Mt. Fanjing observation
Source: Sci Rep. 2026 Jun 10;16:26293. doi: 10.1038/s41598-026-55939-2 (PMC13493816; doi:10.1038/s41598-026-55939-2)
Supplement: Supplementary file 1 [file 41598_2026_55939_MOESM1_ESM.docx]

Supporting Information

**Vertical profile of ambient VOCs in background region of Southwest China from Mt. Fanjing observation**

Dan Yao^1^, Yuan Yang^2*^, Hong Hu^2^, Jingting Yang^2^, Haibo Li^3,4^, Xiangwen Hou^3,4^, Hao Zhang^5^, Xinmin Zhang^6^, Huanjia Liu^1^, Jiaqi Liu^1^, Yanan Teng^1^, Yujuan Fan^1^, Ke Cheng^1^, Jingyun Liu^7^, Yingchao Yan^8^

^1^Key Laboratory of Yellow River and Huai River Water Environment and Pollution Control, Ministry of Education, Henan Key Laboratory for Environmental Pollution Control, School of Environment, Henan Normal University, Xinxiang, Henan, China

^2^Guizhou Research and Designing Institute of Environmental Sciences, Guizhou Academy of Environmental Science and Design, Guiyang, China

^3^Fanjingshan National Nature Reserve Administration, Tongren, Guizhou, China

^4^Guizhou Fanjingshan Forest Ecosystem Observation and Research Station, Tongren, Guizhou, China

^5^School of Public Health, Shandong Second Medical University, Weifang, Shandong, China

^6^State Key Laboratory of Environmental Criteria and Risk Assessment, Chinese Research Academy of Environmental Sciences, Beijing, China

^7^College of Energy and Environmental Engineering, Hebei Key Laboratory of Air Pollution Cause and Impact, Hebei University of Engineering, Handan, 056038, China.

^8^Inner Mongolia Industrial Technology Engineering Center of Special Resources Development, Utilization and Ecological Environment Protection, Inner Mongolia Normal University, Inner Mongolia, 010022, China.

*Correspondence to:* Yuan Yang ([youngyuan2025@163.com](mailto:youngyuan2025@163.com))

**Text S1. Identification of the dominated background winds and local diurnal variation circulation**

**Text S2. VOCs measurements.**

**Text S3. Positive Matrix Factorization (PMF).**

**Text S4. XGBoost model**

**Text S5. The concentration weighted trajectory method**

**Table S1. Selected dates for vertical exchange and valley wind circulation dominated periods.**

**Table S2. Selected dates for the six circulation dominated periods.**

**Table S3. Concentration characteristics of VOCs selected for the PMF and statistics for the different types of VOCs in this study.**

**Table S4. Base run of VOCs selected for the PMF and statistics for the different types of VOCs in this study.**

**Table S5. Mapping of bootstrap factors to base factors.**

**Figure S1.** Time series of VOCs, NO_x_, O_3_ and meteorological factor (T, RH, WD and WS) at the top and foot of Mount Fanjing from November, 2024 to February, 2025.

**Figure S2.** Diurnal variation of VOCs, NOx, CO and meteorological factors **during the observation period at mountain foot (MF) and mountain top (MT) site,** the average sunrise and sunset times during the observation period are 6:30 and 18:30, respectively. The shaded area represents the daytime observation period**.**

Fig.S3 Diurnal variation of T/B and X/E at the mountain top and mountain foot sites during the observation period.

**Figure S4.** Linear regressions of carbonyls between the Foot and Top. Red and green color circles represent daytime and nighttime, respectively.

**Figure S5.** Diurnal variation of boundary layer height (BLH) **during the observation period at both sites (**the data was download by https://cds.climate.copernicus.eu/datasets/reanalysis-era5-single-levels?tab=download**).**

**Figure S6.** Model performance and SHAP feature contributions for TVOCs at the mountain top site: (a) model performance; (b) SHAP feature contributions.

**Figure S7.** Responses of TVOCs at the mountain top and mountain foot sites to temperature and relative humidity.

**Figure S8.** Cluster of air mass trajectory **during the observation period at mountain top (a) and mountain foot (b) site.**

**Figure S9.** Hourly longitude–pressure cross-sections of winds over the Mt. Fanjing region for two typical situations. **(a):** a case without evident mountain–valley circulation, in which the wind direction below 800 hPa remains nearly unchanged throughout the day. **(b):** a case with evident mountain–valley circulation along the 26.5–27.2°N latitude.

**Figure S10.** Diurnal alkanes, alkenes, acetylene and aromatics during a) E-weak, b) E-moderate, c) E-strong, d) W-weak, e) W-moderate, f) W-strong, the average sunrise and sunset times during the observation period are 6:30 and 18:30, respectively. The shaded area represents the daytime observation period.

**Text S1. Identification of the dominated background winds and local diurnal variation circulation**

In this study, we use the ERA5 reanalysis produced by the European Centre for Medium-Range Weather Forecasts (ECMWF) to obtain atmospheric circulation fields over Guizhou Province and the surrounding regions. ERA5 has a horizontal resolution of 0.25° × 0.25°, a temporal resolution of 1 h, and 37 pressure levels from the surface to 1 hPa (Hersbach et al., 2020). Previous evaluation studies have demonstrated that ERA5 reproduces winds and large-scale circulation reasonably well in East Asia, particularly in the mid–lower troposphere (approximately 850–300 hPa), and can reliably capture the East Asian westerly jet, monsoon systems, and circulation associated with the Tibetan Plateau [*Hu et al.*, 2021; *Minola et al.*, 2024]. Therefore, over Guizhou and the southeastern flank of the Tibetan Plateau, ERA5 is suitable for analyzing the background westerlies around 800 hPa and the associated zonal transport along the eastern margin of the Plateau.

It should be noted that Guizhou is characterized by highly complex topography, and the capability of ERA5 to represent near-surface winds and local-scale processes such as valley and slope winds is limited, since such fine-scale circulations are often smoothed at the ERA5 grid scale. Consequently, in this study ERA5 is used only to describe the regional-mean background wind field in the lower troposphere (e.g., around 800 hPa) and the associated zonal transport, and to construct a relative local-circulation index that measures the strength of the day–night wind contrast. We do not attempt to reconstruct detailed valley-wind structures directly from ERA5.

To quantify local circulation characteristics over the Mt. Fanjing region under large-scale background flow, we construct a local-circulation index and define background wind types based on ERA5. Specifically, horizontal winds at approximately 800 hPa are extracted along a longitudinal cross-section covering the study area. Daytime and nighttime winds in the lower troposphere are separately averaged, and the magnitude of their vector difference is computed and then averaged over the longitude band corresponding to the valley of Mt. Fanjing. This yields a local-circulation index that characterizes the strength of the diurnal variation of valley/slope winds. In addition, the background wind vector (u_bg, v_bg) is obtained by averaging the 800 hPa winds over the entire cross-section and over all hours. Based on u_bg, the background flow is categorized into westerly, easterly, and weak-wind types, representing different large-scale regimes of the westerlies and zonal transport along the eastern margin of the Tibetan Plateau affecting Guizhou. Figure S6 presents two typical hourly longitude–pressure cross-sections to illustrate the occurrence and timing of mountain–valley circulation over the Mt. Fanjing region. In the case without evident mountain–valley circulation, the wind direction below 800 hPa remains nearly unchanged throughout the day, indicating the absence of a clear low-level directional transition. By contrast, in the case with evident mountain–valley circulation, a distinct wind-direction change occurs below 800 hPa around 17:00 BJT, suggesting the development of a local circulation-related low-level transition. The intensity of local circulation is evaluated independently using the local-circulation index (LCI), with strong ($LCI>0.6$), moderate ($0.2\leq LCI\leq0.6$), and weak ($LCI<0.2$) categories.

**Text S2. VOCs measurements**

VOCs were measured in real time using an Ntrace VsCMS6500 VOCs automated monitoring system (Ntrace VsCMS6500; Nutech (China) Co.,Ltd., Changsha, China). The instrument employs a unique “dual-column separation + center cutting + single FID” design, enabling comprehensive separation and analysis of VOCs compounds. Ambient air or calibration gas is introduced at a constant flow rate into the sampling system, where interfering substances are removed via cryogenic trapping, followed by enrichment of target analytes. Subsequently, the enriched compounds are thermally desorbed, separated by gas chromatography (GC), and quantified using a hydrogen flame ionization detector (FID). External standard calibration was applied for quantification. A series of standard gases (0.5, 2, 4, 6, 8, and 10 nmol/mol) were analyzed in triplicate from low to high concentrations. Calibration curves were constructed using least-squares regression, with target compound concentrations as the abscissa and peak areas as the ordinate. The correlation coefficients (R²) of all calibration curves were ≥0.98. The relative error between the measured and reference values at the lowest calibration point was ≤15%. Method detection limit (MDL): ≤0.1 nmol/mol for 90% of the species. In summary, the system enables continuous sampling, processing, and detection of ambient VOCs, providing reliable quantification of 57 photochemical precursors.

**Text S3. Positive Matrix Factorization (PMF)**

In this study, we used EPA PMF 5.0 to identify the possible sources of ambient VOCs. According to the selection principles of the input datasets provided in previous studies (Li et al., 2019; Liu et al., 2017), 1848 hourly samples involving 22 species (including 11 alkanes, 4 alkenes, 6 aromatics and acetylene) were applied to the PMF model. The corresponding uncertainties of the input datasets were calculated by Equations (1) and (2) where MDL represents the method detection limit, and the error fraction was set to 10%.

$Unc=\frac{5}{6}\times MDL Concentration\leq MDL$ (1)

$Unc=\sqrt{{(ErrorFraction\times Con)}^{2}+{(0.5\times MDL)}^{2}} Concentration\geq MDL$ (2)

Eventually, Modeling was performed for 5-8 factors, and the 6-factor solution was deemed to be the most representative. With 6 factors, more than 96% of the scaled residuals of the chemical species for the selected results are distributed between -3 to +3. The modeled VOCs fit well with measured total VOCs with the correlation coefficient (R^2^) of 0.99, suggesting the observed VOC concentrations were well represented by the PMF model-resolved source factors at Mt. Fanjing. Bootstrapping mapping of PMF factor solutions are shown in Table S5, indicating the results were more stable with all factors mapped in BS is more than 80%, and there are no unmapped BS factors and no swaps with DISP. To further determine if source contributions are really different, concentration characteristics of VOCs selected for the PMF and statistics for the different types of VOCs and base run of VOCs selected for the PMF and statistics for the different types of VOCs were provided in Table S3, and the base run of VOCs selected for the PMF and statistics for the different types of VOCs in Mt. Fanjing were shown in Table S4. In summary, the analysis of uncertainty revealed that the identified source profiles are reasonable in this study.

During the observation period, Gasoline vehicle-related &biomass burning accounted for larger percentages of isopentane (40.8%) and acetylene (68.5%); Industry-related contained a high proportion of benzene (43.8%), toluene (24.7%), ethylbenzene (45.3%), xylene (45.9%), ethene (41.7%) and styrene (38.9%). Chemical production was characterized by high values of propene (43.5%) and 1-butene (68.5%); Solvent usage accounted for large amount of toluene (32.9%), xylene (33.4%) ethylbenzene (33.8%) and hexane (22.1%); Liquefied petroleum gas and natural gas (LPG&NG) was characterized by high values of C_2_-C_4_ alkanes (29.0%~44.7%); Aged traffic plume contained a high proportion of n-decane (76.5%) and C_6_~C_7_ alkanes (29.0%~34.9%).

**Text S4. XGBoost model**

This study employed the XGBoost model to predict and validate TVOCs.MT, and the results were shown in Figure S6a. Overall, the predicted values exhibited good linear agreement with the measured values. Most sample points were distributed close to the 1:1 reference line, indicating that the model had strong fitting and predictive capability. In the training set, the coefficient of determination (R²) was 0.971, the RMSE was 0.996, and the MAE was 0.705, suggesting that the model had a high explanatory power for the training data and relatively small prediction errors. In the test set, the R² was 0.770, the RMSE was 2.651, and the MAE was 1.742 （Fig.S5a）. Although the prediction accuracy declined compared with that of the training set, the model still maintained good overall generalization ability. The performance gap between the training and test sets indicates that the model exhibited a certain degree of overfitting, but this remained within an acceptable range.

From the characteristics of the scatter distribution, the model performed relatively stably in the low- and medium-concentration ranges, where the sample points aligned well with the 1:1 line. In contrast, the scatter became more dispersed in the high-concentration range, indicating increased prediction errors for high-value samples. This may be related to the relatively small number of high-concentration samples, the right-skewed data distribution, and the more complex variation patterns in the high-value region. The marginal distribution plots also showed that both the observed and predicted values exhibited clear skewed distribution characteristics, further suggesting that the model was more effective at learning patterns in densely distributed sample ranges, while its ability to characterize tail-end samples was relatively limited.

The residual analysis showed that most residuals were randomly distributed around 0, indicating that the model had no obvious systematic bias overall. However, as the predicted values increased, the range of residual fluctuations also widened, revealing a certain degree of heteroscedasticity. In addition, a few outliers were observed, suggesting that the model’s stability in the high-prediction-value region still has room for improvement. Overall, the XGBoost model was able to predict TVOCs.MT with relatively high accuracy and demonstrated good fitting performance as well as practical application potential. Nevertheless, its generalization performance and prediction accuracy in the high-value range could be further improved by increasing the number of high-value samples, optimizing feature variables, or adjusting model parameters”.

**Text S5. The concentration weighted trajectory method**

CWT is typically used to identify potential source regions of air pollutants by combining pollutant concentrations measured at the receptor with air-mass back-trajectory residence times, thereby highlighting upwind areas associated with stronger contributions. In the CWT method, each grid cell is assigned a weighted concentration by averaging the sample pollutant concentrations that have associated trajectories crossing the grid cell as follows: where $C_{ij}$is the mean weighted concentration in the $ij^{th}$cell, $l$is the index of the trajectory, $C_{l}$is the pollutant concentration measured on the arrival of trajectory $l$, $M$is the total number of trajectories, and $\tau_{ijl}$is the time spent in the $ij^{th}$cell by trajectory $l$. In the CWT analysis, the above arbitrary weighting function is also used to reduce the influence of the small $n_{ij}$value in the PSCF model.

$$\text{C}\text{ij}\text{=}\frac{\text{1}}{\sum_{\text{l=1}}^{\text{M}} \text{τ}\text{ijl}}\sum_{\text{l=1}}^{\text{M}} \text{τ}\text{ijl}$$

**Table S1.** Selected dates for vertical exchange and valley wind circulation dominated periods.

| Species | Top^1^:  2119 m | Species | (Foot^1^:  550 m) | Species | Qin  Ling^2^ | Species | Xing  long^3^ | Species | Dinghushan^4^ |
| --- | --- | --- | --- | --- | --- | --- | --- | --- | --- |
| ethane | 3.4 | ethane | 4.2 | Propane | 2.6 | Ethane | 2.5 | Toluene | 2.3 |
| acetylene | 1.8 | propane | 2.2 | n-Butane | 2.5 | Acetone | 2.2 | Benzene | 1.4 |
| propane | 1.8 | acetylene | 1.8 | Ethane | 1.5 | Propane | 1.3 | 3-methyl-hexane | 1.4 |
| ethene | 0.7 | ethene | 1.6 | Iso-butane | 1.5 | Acetylene | 0.8 | Isopentane | 1.3 |
| m,p-xylene | 0.7 | n-butane | 0.7 | Ethylene | 0.9 | n-Butane | 0.5 | n-Butane | 1.3 |
| 1,2,3-trimethylbenzene | 0.5 | isobutane | 0.5 | n-Pentane | 0.5 | Ethylene | 0.5 | 2,3-dimethyl-pentane | 1.3 |
| n-butane | 0.5 | benzene | 0.5 | Iso-Pentane | 0.4 | Benzene | 0.4 | 2-methyl-hexane | 1.1 |
| 1,2,4-trimethylbenzene | 0.5 | m,p-xylene | 0.5 | 1-Butene | 0.3 | i-Butane | 0.3 | Ethylbenzene | 1.0 |
| n-propylbenzene | 0.5 | n-decane | 0.5 | Propylene | 0.2 | i-Pentane | 0.3 | Butene | 0.9 |
| benzene | 0.4 | 1,3,5-trimethylbenzene | 0.5 | Acetylene | 0.2 | Toluene | 0.2 | 2-methyl-Pentane | 0.8 |
| styrene | 0.4 | m-ethyltoluene | 0.4 | Benzene | 0.2 | n-Pentane | 0.2 | Isoprene | 0.8 |
| n-decane | 0.4 | o-ethyltoluene | 0.4 | Toluene | 0.1 | n-Hexane | 0.1 | 3-methyl-pentane | 0.7 |

Note:^1^this study, ^2^ Xue et al., 2023; ^3^Yao et al., 2022; ^4^Wu et al., 2016.

**Table S2.** Selected dates for the six circulation dominated periods.

| Periods | Dates |
| --- | --- |
| E-weak | 2024: 11/2, 12/7;  2025:1/6, 1/7, 2/5, 2/7 |
| E-moderate | 2024: 11/1, 11/4, 11/5, 11/7, 11/8, 11/16-11/21,11/27, 12/6, 12/8, 12/11, 12/18, 12/21, 12/22, 12/27, 12/28;  2025: 1/26, 1/28, 2/2, 2/3 |
| E-strong | 2024: 11/6,11/26, 12/3,12/5, 12/14, 12/31;  2025: 1/5, 1/9-1/12,1/15, 1/17, 2/4, 2/6,2/8, 2/12, 2/13 |
| W-weak | 2024: 11/9, 11/10, 11/24, 11/30, 12/2, 12/30;  2025: 1/16, 1/19, 2/10, 2/15 |
| W-moderate | 2024: 11/3, 11/11,11/13, 11/14, 11/23, 11/29, 12/10, 12/16;  2025: 1/3, 1/14, 1/20-1/23, 1/29, 1/31, 2/14 |
| W-strong | 2024: 11/12, 11/25, 12/4, 12/9, 12/15, 12/19, 12/20, 12/23, 12/24, 12/29  2025: 1/4, 1/8, 1/18, 1/24, 1/30, 2/11 |

**Table S3.** Concentration characteristics of VOCs selected for the PMF and statistics for the different types of VOCs in this study.

| Species | Category | S/N | Min | 25th | Median | 75th | Max | % Modeled Samples |
| --- | --- | --- | --- | --- | --- | --- | --- | --- |
| ethane | Strong | 10.0 | 1.3 | 2.9 | 3.8 | 4.5 | 8.9 | 98.68% |
| propane | Strong | 10.0 | 0.5 | 1.3 | 1.9 | 2.4 | 5.7 | 98.68% |
| n-butane | Strong | 8.8 | 0.2 | 0.3 | 0.4 | 0.5 | 1.1 | 98.68% |
| isobutane | Strong | 9.5 | 0.2 | 0.5 | 0.6 | 0.8 | 1.8 | 98.68% |
| isopentane | Strong | 5.8 | 0.1 | 0.2 | 0.2 | 0.3 | 0.7 | 98.68% |
| n-pentane | Strong | 6.4 | 0.1 | 0.2 | 0.2 | 0.3 | 0.5 | 98.68% |
| n-hexane | Strong | 4.7 | 0.1 | 0.1 | 0.2 | 0.2 | 0.3 | 98.68% |
| 3-methylhexane | Strong | 3.9 | 0.1 | 0.1 | 0.1 | 0.1 | 0.2 | 98.68% |
| n-heptane | Strong | 3.7 | 0.1 | 0.1 | 0.1 | 0.2 | 0.2 | 98.68% |
| 2_3_4 - Trimethylpentane | Strong | 3.8 | 0.1 | 0.1 | 0.1 | 0.2 | 0.2 | 98.68% |
| n-dodecane | Strong | 5.1 | 0.1 | 0.1 | 0.3 | 0.3 | 0.4 | 98.68% |
| ethene | Strong | 9.8 | 0.2 | 0.7 | 1.1 | 1.6 | 3.6 | 98.68% |
| propene | Strong | 5.4 | 0.1 | 0.1 | 0.2 | 0.3 | 0.5 | 98.68% |
| 1-butene | Strong | 5.4 | 0.1 | 0.1 | 0.2 | 0.3 | 0.6 | 98.68% |
| cis-2-butene | Strong | 3.3 | 0.1 | 0.1 | 0.1 | 0.1 | 0.2 | 98.68% |
| acetylene | Strong | 9.6 | 0.1 | 0.9 | 1.4 | 2.0 | 9.3 | 98.68% |
| benzene | Strong | 9.1 | 0.2 | 0.4 | 0.5 | 0.5 | 1.2 | 98.68% |
| toluene | Strong | 7.2 | 0.1 | 0.2 | 0.3 | 0.3 | 0.6 | 98.68% |
| ethylbenzene | Strong | 6.4 | 0.1 | 0.2 | 0.3 | 0.3 | 0.6 | 98.68% |
| m,p-xylene | Strong | 9.4 | 0.2 | 0.5 | 0.6 | 0.6 | 1.7 | 98.68% |
| styrene | Strong | 8.4 | 0.1 | 0.4 | 0.4 | 0.5 | 0.5 | 98.68% |
| o-xylene | Strong | 7.0 | 0.1 | 0.3 | 0.3 | 0.3 | 0.7 | 98.68% |

**Table S4.** Base run of VOCs selected for the PMF and statistics for the different types of VOCs in this study.

| Species | Intercept | Slope | SE | r^2 | Stat | P Value |
| --- | --- | --- | --- | --- | --- | --- |
| ethane | 0.01 | 0.99 | 0.30 | 0.95 | 0.08 | 0.21 |
| propane | 0.09 | 0.95 | 0.12 | 0.99 | 0.05 | 0.80 |
| n-butane | 0.08 | 0.81 | 0.07 | 0.80 | 0.12 | 0.01 |
| isobutane | 0.02 | 0.97 | 0.04 | 0.98 | 0.07 | 0.31 |
| isopentane | 0.03 | 0.85 | 0.03 | 0.87 | 0.04 | 0.95 |
| n-pentane | 0.06 | 0.73 | 0.03 | 0.80 | 0.09 | 0.08 |
| n-hexane | 0.02 | 0.90 | 0.02 | 0.76 | 0.06 | 0.62 |
| 3-methylhexane | 0.03 | 0.77 | 0.01 | 0.82 | 0.11 | 0.03 |
| n-heptane | 0.00 | 0.98 | 0.01 | 0.94 | 0.09 | 0.09 |
| 2,3,4 - Trimethylpentane | 0.00 | 0.98 | 0.01 | 0.92 | 0.07 | 0.37 |
| n-dodecane | 0.00 | 1.00 | 0.03 | 0.96 | 0.04 | 0.87 |
| ethene | 0.12 | 0.88 | 0.15 | 0.93 | 0.05 | 0.70 |
| propene | 0.01 | 0.93 | 0.03 | 0.92 | 0.06 | 0.49 |
| 1-butene | 0.01 | 0.92 | 0.03 | 0.96 | 0.07 | 0.32 |
| cis-2-butene | 0.03 | 0.69 | 0.01 | 0.56 | 0.16 | 0.00 |
| acetylene | 0.00 | 0.99 | 0.21 | 0.98 | 0.09 | 0.15 |
| benzene | 0.10 | 0.76 | 0.04 | 0.85 | 0.07 | 0.30 |
| toluene | 0.13 | 0.52 | 0.03 | 0.65 | 0.06 | 0.59 |
| ethylbenzene | 0.02 | 0.91 | 0.02 | 0.93 | 0.10 | 0.06 |
| m,p-xylene | 0.16 | 0.70 | 0.07 | 0.77 | 0.19 | 0.00 |
| styrene | 0.00 | 1.00 | 0.03 | 0.93 | 0.09 | 0.09 |
| o-xylene | 0.03 | 0.89 | 0.03 | 0.90 | 0.11 | 0.03 |

**Table S5.** Mapping of bootstrap factors to base factors.

|  | Factor 1 | Factor 2 | Factor 3 | Factor 4 | Factor 5 | Factor 6 | Unmapped |
| --- | --- | --- | --- | --- | --- | --- | --- |
| Boot Factor 1 | 100 | 0 | 0 | 0 | 0 | 0 | 0 |
| Boot Factor 2 | 1 | 98 | 1 | 0 | 0 | 0 | 0 |
| Boot Factor 3 | 1 | 2 | 96 | 0 | 0 | 1 | 0 |
| Boot Factor 4 | 10 | 2 | 1 | 84 | 0 | 3 | 0 |
| Boot Factor 5 | 0 | 0 | 0 | 0 | 100 | 0 | 0 |
| Boot Factor 6 | 0 | 2 | 0 | 0 | 0 | 98 | 0 |

**Figure S1.** Time series of VOCs, NO_x_, O_3_ and meteorological factor (T, RH, WD and WS) at the top and foot of Mount Fanjing from November, 2024 to February, 2025.

**Figure S2.** Diurnal variation of VOCs, NOx, CO and meteorological factors **during the observation period at mountain foot (MF) and mountain top (MT) site,** the average sunrise and sunset times during the observation period are 6:30 and 18:30, respectively. The shaded area represents the daytime observation period**.**

Fig.S3 Diurnal variation of T/B and X/E at the mountain top and mountain foot sites during the observation period.

**Figure S4.** Linear regressions of carbonyls between the Foot and Top. Red and green color circles represent daytime and nighttime, respectively.

**Figure S5.** Diurnal variation of boundary layer height (BLH) **during the observation period at both sites (**the data was download by https://cds.climate.copernicus.eu/datasets/reanalysis-era5-single-levels?tab=download**).**

**Figure S6.** Model performance and SHAP feature contributions for TVOCs at the mountain top site: (a) model performance; (b) SHAP feature contributions.

**Figure S7.** Responses of TVOCs at the mountain top and mountain foot sites to temperature and relative humidity.

**Figure S8.** Cluster of air mass trajectory **during the observation period at mountain top (a) and mountain foot (b) site.**

a)

b)

**Figure S9.** Hourly longitude–pressure cross-sections of winds over the Mt. Fanjing region for two typical situations. **(a):** a case without evident mountain–valley circulation, in which the wind direction below 800 hPa remains nearly unchanged throughout the day. **(b):** a case with evident mountain–valley circulation along the 26.5–27.2°N latitude.

**Figure S10.** Diurnal alkanes, alkenes, acetylene and aromatics during a) E-weak, b) E-moderate, c) E-strong, d) W-weak, e) W-moderate, f) W-strong, the average sunrise and sunset times during the observation period are 6:30 and 18:30, respectively. The shaded area represents the daytime observation period.

**References**

Hersbach, H., Bell, B., Berrisford, P., Hirahara, S., Horányi, A., Muñoz-Sabater, J., Nicolas, J., Peubey, C., Radu, R., Schepers, D., Simmons, A., Soci, C., Abdalla, S., Abellan, X., Balsamo, G., Bechtold, P., Biavati, G., Bidlot, J., Bonavita, M., Chiara, G.D., Dahlgren, P., Dee, D., Diamantakis, M., Dragani, R., Flemming, J., Forbes, R., Fuentes, M., Geer, A., Haimberger, L., Healy, S., Hogan, R.J., Hólm, E., Janisková, M., Keeley, S., Laloyaux, P., Lopez, P., Lupu, C., Radnoti, G., Rosnay, P., Rozum, I., Vamborg, F., Villaume, S., Thépaut, J.-N. The ERA5 global reanalysis. Q J R Meteorol Soc. 2020; 146: 1999–2049. 10.1002/qj.3803.

Hsu, Y.K., Holsen, T. M., & Hopke, P. K. *Comparison of hybrid receptor models to locate PCB sources in Chicago*. Atmospheric Environment, 2003, 37: 545–562. 10.1016/S1352-2310(02)00886-5.

Hu, H., Cao, Y.C., Shi, C., Lei, Y., Wen, H., Liang, H., Tu, M.H., Wan, X.M., Wang, H.S., Liang, J.S. Analysis of the Precipitable Water Vapor Observation in Yunnan-Guizhou Plateau during the Convective Weather System in Summer, Atmos., 2021, 12: 1085. 10.3390/atmos12081085.

Li, B.W., Ho, S.S.H., Gong, S.L., Ni, J.W., Li, H.R., Han, L.Y., Yang, Y., Qi, Y.J., Zhao, D.X., 2019. Characterization of VOCs and their related atmospheric processes in a central Chinese city during severe ozone pollution periods. Atmos. Chem. Phys. 19, 617-638. 10.5194/acp-19-617-2019.

Liu, C.T., Ma, Z.B., Mu, Y.J., Liu, J.F., Zhang, C.L., Zhang, Y.Y., Liu, P.F., Zhang, H.X., 2017. The levels, variation characteristics, and sources of atmospheric non-methane hydrocarbon compounds during wintertime in Beijing, China. Atmos. Chem. Phys. 17, 10633-10649. [10.5194/acp-17-10633-2017](http://doi.org/10.5194/acp-17-10633-2017).

Minola, L., Zhang, G.F., Ou, T.H., Kukulies, J.L., Curio, J.L., Guijarro, J.A., Deng, K.Q., Azorin-Molina, C., Shen, C., Pezzoli, A., Chen, D.L. Climatology of near-surface wind speed from observational, reanalysis and high-resolution regional climate model data over the Tibetan Plateau. Clim. Dyn., 2024, 62: 933–953. [10.1007/s00382-023-06931-3](https://doi.org/10.1007/s00382-023-06931-3).

Wu, F.K., Yu, Y., Sun, J., Zhang, J.K., Wang, J., Tang, G.Q., Wang, Y.S. Characteristics, source apportionment and reactivity of ambient volatile organic compounds at Dinghu Mountain in Guangdong Province, China, Sci. Total Environ., 2016, 548, 347-359. 10.1016/j.scitotenv.2015.11.069.

Xue, Y.G., Wang, L.Q., Jing, Y., Zhang, T., He, S., Ho, K.F., Huang, Y., Chen, L., Cui, L., and Cao, J.J.: Asian Monsoon and Local Valley Wind Caused Transport of Volatile Organic Compounds Episode Across Qinling Mountain in China, J GEOPHYS RES-ATMOS, 2023, 128, e2022JD038256, 10.1029/2022jd038256.

Yao, D., Tang, G.Q., Wang, Y.H., Yang, Y., Wang, Y.M., Liu, Y.S., Yu, M., Liu, Y.T., Yu, H., Liu, J.D., Hu, B., Wang, P.C., and Wang, Y.S.: Oscillation cumulative volatile organic compounds on the northern edge of the North China Plain: Impact of mountain-plain breeze, Sci. Total Environ., 2022, 821, 153541, 10.1016/j.scitotenv.2022.153541.
